# A practical strategy for responding to a case of lymphatic filariasis post-elimination in Pacific Islands

**DOI:** 10.1186/1756-3305-6-218

**Published:** 2013-07-26

**Authors:** Humpress Harrington, James Asugeni, Christopher Jimuru, John Gwalaa, Elmer Ribeyro, Richard Bradbury, Hayley Joseph, Wayne Melrose, David MacLaren, Rick Speare

**Affiliations:** 1 Atoifi Adventist Hospital, Malaita, Solomon Islands; 2 Medical Research Council International Nutrition Group, MRC, Keneba, The Gambia; 3 WHO Collaborating Centre for the Control of Lymphatic Filariasis and Soil Transmitted Helminths, School of Public Health, Tropical Medicine and Rehabilitation Sciences, James Cook University, Townsville, Australia; 4 School of Medicine and Dentistry, James Cook University, Cairns, Australia; 5 Tropical Health Solutions Pty Ltd, Townsville, Australia

**Keywords:** Lymphatic filariasis, Elimination, Solomon Islands, Atoifi, *Wuchereria bancrofti*, Surveillance response, Research capacity building

## Abstract

**Background:**

Lymphatic filariasis (LF) due to *Wuchereria bancrofti* is being eliminated from Oceania under the Pacific Elimination of Lymphatic Filariasis Programme. LF was endemic in Solomon Islands but in the 2010-2020 Strategic Plan of the Global Programme to Eliminate LF, Solomon Islands was listed as non-endemic for LF. In countries now declared free of LF an important question is what monitoring strategy should be used to detect any residual foci of LF?

This paper describes how a new case of elephantiasis in a post-elimination setting may be used as a trigger to initiate a local survey for LF.

**Methods:**

The index case, a 44 year old male, presented to Atoifi Adventist Hospital, Malaita, Solomon Islands in April 2011 with elephantiasis of the lower leg. Persistent swelling had commenced 16 months previously. He was negative for antigen by TropBio Og4C3 ELISA and for microfilaria. A week later a survey of 197 people aged from 1 year to 68 years was conducted at Alasi, the index case’s village, by a research team from Atoifi Adventist Hospital and Atoifi College of Nursing. This represented 66.3% of the village population. Blood was collected between 22:00 and 03:00 by finger-prick and made into thick smears to detect microfilaria and collected onto filter paper for *W. bancrofti* antigen tests. A second group of 110 specimens was similarly collected from residents of the Hospital campus and inpatients. *W. bancrofti* antigen was tested for using the Trop-Bio Og4C3 test.

**Results:**

One sample (1/307) from an 18 year old male from Alsai was positive for *W. bancrofti* antigen. No samples were positive for microfilaria. Although antigen-positivity indicated a live worm, the case was regarded as having been acquired some years previously.

**Conclusions:**

We propose that when LF has been eliminated from a country, a case of elephantiasis should be a trigger to conduct a survey of the case’s community using a decision pathway. *W. bancrofti* antigen should be tested for with screening for microfilariae in antigen positive cases. The field survey was designed and conducted by local researchers, highlighting the value of local research capacity in remote areas.

## Background

Lymphatic filariasis (LF) is of global concern as a major cause of morbidity in many tropical countries. Following the success of eliminating the parasite from industrialized countries in its former range, the World Health Assembly approved a global elimination programme [1]. The Pacific Programme for the Elimination of Lymphatic Filariasis (PacELF) was formed in 1999 to coordinate the control efforts in Pacific Island Countries and Territories (PICTs) within the framework of the Global Programme to Eliminate Lymphatic Filariasis [2]. The WHO target is to eliminate LF as a public health problem, meaning that the parasite may continue to circulate at a very low level but new cases of clinical disease are unlikely to arise. The elimination target is less than 1/10,000 cases in the population [3].

PacELF and the PICTs are advised and assisted technically in the elimination programme by the WHO Collaborative Centre for Control of Lymphatic Filariasis and Soil Transmitted Helminths based at James Cook University, Townsville. The main mechanism used by the elimination programme to stop transmission of LF is community wide administration of oral albendazole and diethylcarbamazine every 12 months [4]. Of the 22 countries within the PICTs LF was classed as wholly or partly endemic in 17. Fourteen of the endemic countries have implemented Mass Drug Administration programmes [5]. When LF is close to elimination, it appears to persist in countries as small geographic foci centred around individual(s) with microfilaremia [6,7]. The surveillance strategy proposed for the Pacific in the final stage of the LF elimination programme is based on using children as sentinels for ongoing transmission by detection of antigen of *W. bancrofti* in the blood. Finding a positive child will then initiate “close contact testing” in which surrounding households are tested [8]. This strategy relies on ongoing surveys of children for antigen. It is feasible that LF could persist in remote locations too logistically demanding to survey by researchers from national programmes based in capital cities.

Solomon Islands, a PICT with a population of 525,000, distributed over 8 provinces, has eliminated LF, possibly as a spin-off of a very active malaria eradication programme pre-1985 [9,10]. In a survey conducted using thick blood films to detect microfilaria in the early 1940s the prevalence of LF in the Solomon Islands was 19.6%, with the prevalence in Malaita (the province dealt with in this paper) being 10.2% [11]. By 1976 prevalence in Choiseul province had declined to 5.5% [11] and to 2% in Guadacanal province [9]. In Choiseul province the prevalence of mf positivity had fallen to zero by 1978 associated with spraying for malaria control [10]. Microfilaremia was still present in other provinces [10]. In 2002 a survey of primary school children for antigen detected by ICT cards found no positive cases [12]. In the 2010-2020 strategic plan of the Global Programme to Eliminate Lymphatic Filariasis, Solomon Islands was considered non-endemic for LF [5]. This conclusion was based on the opinions of a global working group of experts, who reviewed all available evidence [13] although no data on prevalence appears to have been published for 30 years.

In a country that has reached the verification stage of LF elimination expected surveillance activities include (among other strategies) a review of filariasis case reports through routine disease surveillance or other systems for case detection plus a description of case follow-up activities for each positive case detected [14]. No criteria have been proposed for countries that had LF in the past, did not participate in the recent elimination programme, but are now regarded as non-endemic for LF. Solomon Islands is such a country.

Here we describe an instance in the Solomon Islands where a new case of elephantiasis in East Kwaio, a remote location on the east coast of the island of Malaita, was used as a trigger to conduct a village based survey for LF. The rapid response was conducted by a local team of researchers based in this remote area and trained in operational research techniques.

## Methods

**Figure 1:**
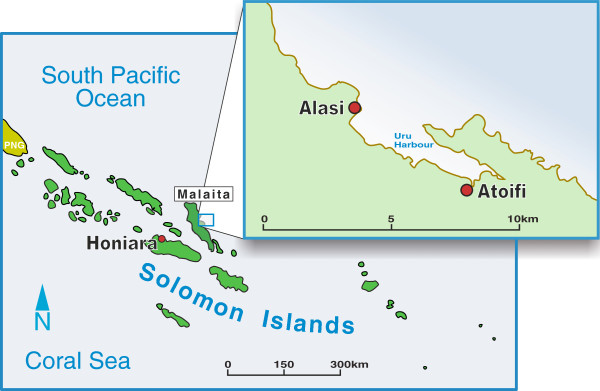
**Map.** Map of Solomon Islands showing location of area of interest with a larger map of Uru Harbour showing Atoifi and Alasi.

Location: Malaita is a predominantly rural island to the North East of Honiara, the capital of Solomon Islands, with a population of 137,600. East Kwaio is on the remote east coast of Malaita. Atoifi Adventist Hospital was established in 1966 in East Kwaio and provides health care, including surgery, in-patient and out-patient services to the surrounding villages (8°52’11”S 151°00’14”E) (Figure 1). Access to Atoifi is via small plane (twice weekly) or boat. The village of Alasi, the location of the index case, is a small isolated village (8°51’58”S, 160°59’41”E), approximately 6 km north of Atoifi, accessible by canoe across Uru Harbour (Figure 1).

### Clinical case of elephantiasis

**Figure 2:**
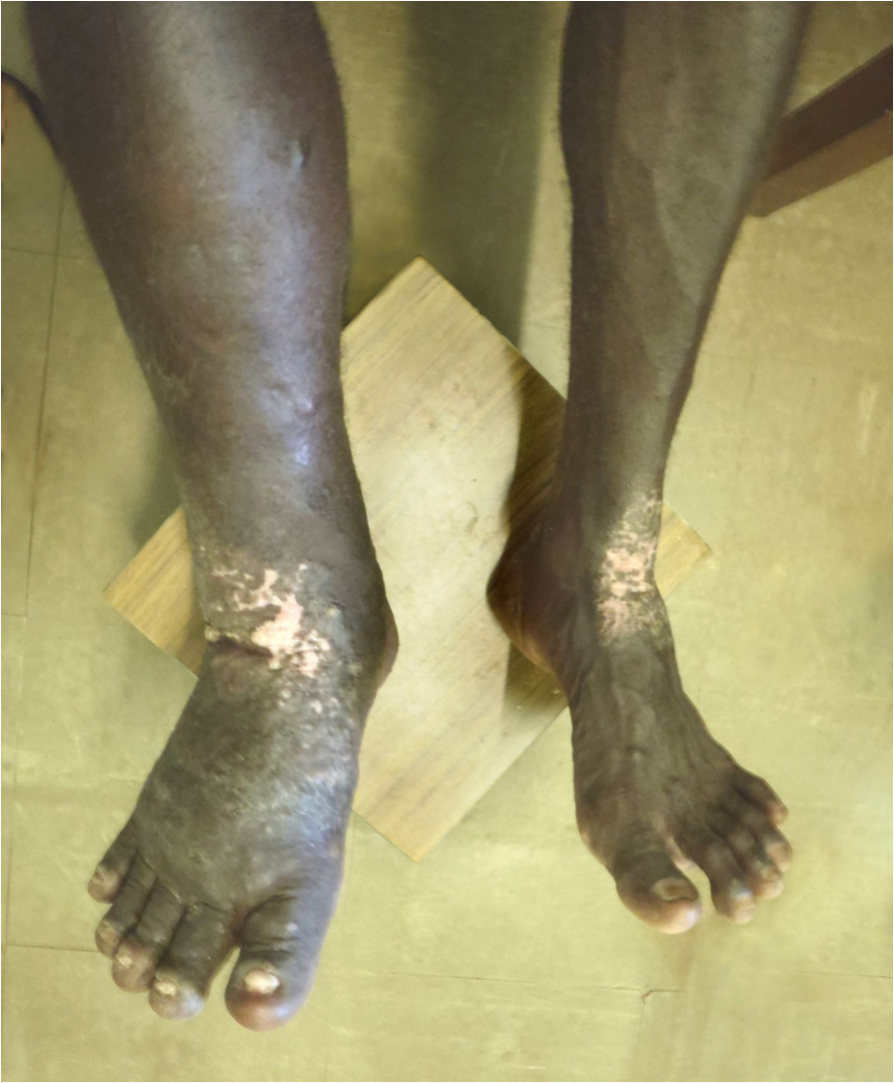
**Both legs (anterior).** Male 44 years from Alasi, Malaita, Solomon Islands with swelling of right lower leg of 16 months duration. Vitiligo is present on flexor aspects of both ankles and had appeared before the limb swelling.

**Figure 3:**
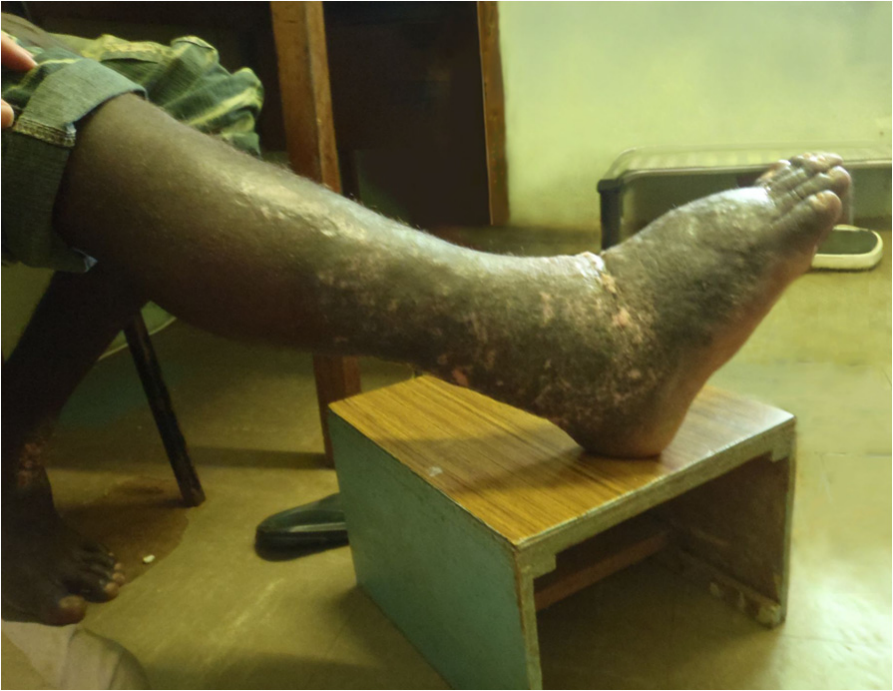
**Right leg (lateral).** Elephantiasis of right leg of 44 year old male from Alasi, Malaita, Solomon Islands (lateral view).

**Figure 4:**
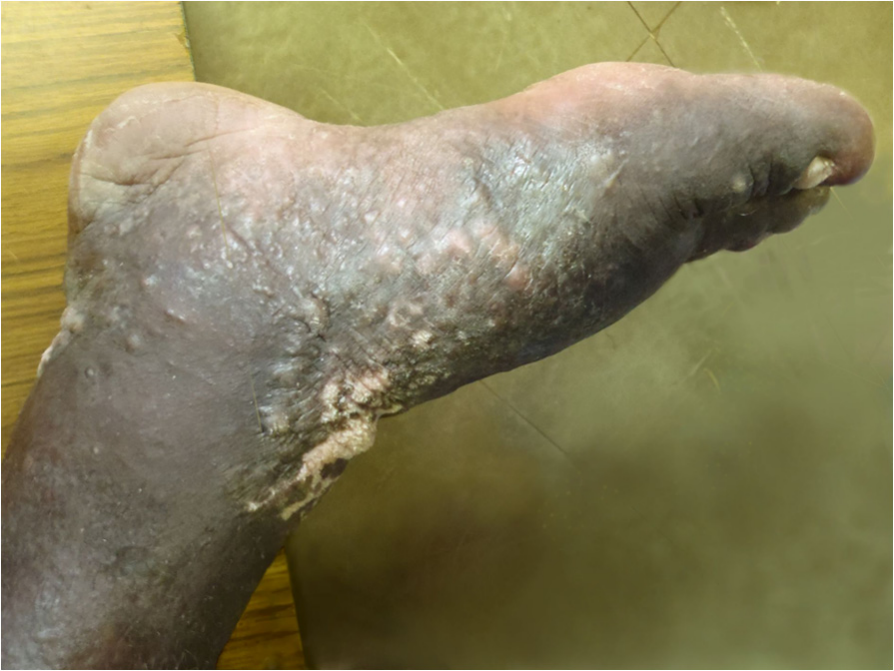
**Skin lesions foot.** Nodular epidermal changes associated with elephantiasis of right leg (medial view).

On 11 April 2011 a 44 year old male presented at Atoifi Adventist Hospital with a 16 month history of swelling of his right lower leg, involving the foot, ankle and the distal two-thirds of the tibia (Figures 2 and 3). The swelling was painless, but the patient complained of heaviness of the limb. Subcutaneous edema was demonstrated in all areas by pitting on digital pressure for 15 seconds. On the foot and ankle subcutaneous tissue was increased markedly and felt dense to palpation, indicating fibrosis. The overlying skin was thickened and covered with low, dome shaped, smooth nodular lesions up to 5 mm in diameter. On the foot these became almost confluent (Figure 4). The foot was warm to touch. An irregular area of vitiligo was present on the flexor surface of the ankle. However, this was matched by a similar patch of vitiligo on the left ankle which was unaffected by swelling (Figure 2). Inguinal lymph nodes on the right were enlarged, but not tender. The patient was afebrile with normal cardiac and respiratory rates. The clinical diagnosis was elephantiasis Grade IV, the grading being based on persistent, irreversible swelling due to increased subcutaneous tissue with knobs and lumps forming on the skin [15].

Medical records showed that the limb was normal in April 2009 and that the patient had first presented with swelling of the right foot on 15 February 2010. The patient was seen again on 24 Feb 2010 and 14 Feb 2011 with acute episodes of tenderness and swelling. At these presentations the focus was on the clinical management of the case.

### Public health implications

Although Solomon Islands had been an LF endemic country in the past, in 2011 it was considered to be free of lymphatic filariasis. The case of recent elephantiasis therefore raised the possibility that transmission of LF may still be occurring in this remote area. Prior to April 2011 the patient had been seen purely as a clinical case and the public health implications had been ignored. However, once the local health professionals were made aware of the programmatic significance of the case, they were adamant that the index case’s community be investigated for active transmission of *W. bancrofti*.

### Immediate response

Index case. The patient was given oral doxycycline, soap to wash the limb and advice on care. The patient and his wife were asked to attend the hospital at night to enable a nocturnal blood collection for testing for microfilaria. Blood was collected via venipuncture at 23:00 since in the Solomon Islands *W. bancrofti* has nocturnal periodicity [16]. Duplicate Knott’s concentration tests, examination of fresh unstained wet thick film (0.05 ml) and Giemsa stained thick film for malaria (0.02 ml) was performed on EDTA blood in the Atoifi Hospital Laboratory for both the patient and his wife. No microfilaria were detected in either. Serum was subsequently transported to Townsville, Australia and tested for *W. bancrofti* antigen using the TropBio Og4C3 ELISA.

### Community survey

The possibility of ongoing transmission of LF at Alasi was presented in a formal lecture at Atoifi Hospital two days later by author RS. The lecture was given during a research symposium taking place at the hospital on 14 April 2011 to conclude a two week research capacity building workshop [17]. The need to assess whether LF transmission was current was then discussed with the local research group at the Hospital. Owing to the nature of the research training delivered, the research group consisted of health professionals (nurses, doctor, and pharmacist) and community leaders (village chiefs, pastors, teachers) all from East Kwaio [17,18]. No one in the group was from Alasi, but several members had relatives who lived in the village.

The group decided that the situation warranted a community survey to investigate the research hypothesis that “Community members in Alasi village (particularly adults) are infected with *Wuchereria bencrofti* and will have microfilaremia”. From this point the field-based survey was managed by the local group (all expatriats having departed Atoifi).

Local research team representatives firstly set out to talk to community people in Alasi village and to residents of the Atoifi Hospital campus (most hospital employees and their families reside on the hospital campus). The Alasi community decided that the survey was valuable and agreed to participate.

Blood was collected between 22:00 hours on 19 April 2011 to 03:00 on 20 April 2011 during a cross-sectional survey of the residents of Alasi. At this stage travel histories were of no importance since the aim was to collect specimens to enable LF to be ruled out. Residents from houses surrounding the house of the index case were given priority since the number of filter papers was limited. Finger prick was used and blood absorbed for each person onto at least 3 filter paper ears, capable of holding 100 μl each. From each participant two thick smears were made on glass microscope slides. Two techniques were used to test for LF (see Laboratory Techniques).

As an additional group residents of the Atoifi Adventist Hospital campus and some inpatients from surrounding villages were also screened in a similar way between 22:00 on 20 April 2011 and 03:00 on 21 April 2011.

### Laboratory techniques

A two step screening process was used: firstly, serum eluted from the filter paper ears was screened using the TropBio Og4C3 ELISA to detect *W. bancrofti* antigen. Secondly, any films of participants with positive or borderline antigen results were examined. As a quality control measure 10% of smears from antigen negative participants were also examined.

The Og4C3 ELISA was performed as previously described and as per manufacturer’s instructions [19]. All thick smears were dried and stained unfixed with a standard Giemsa stain. Stained films were examined using a compound light microscope and the ×40 objective.

### Ethics

Ethics approval was granted for the community survey by the Atoifi Adventist Hospital Research Ethics Committee (AAHREC4) and James Cook University Human Research Ethics Committee (H4002).

### Consent

Written informed consent was obtained from the patient for the publication of this report and any accompanying images.

### Capacity building component

The opportunity to conduct this survey came at the conclusion of a two week research capacity building workshop held at Atoifi that had trained local health professionals and community leaders in how to design and conduct applied health research projects [17,18]. The survey was designed as a collaborative process with the diverse research group. Limited specific LF training was given, including theory (the parasite and its life cycle, disease manifestations, diagnosis, and significance of finding elephantiasis in a LF-free region) and practical training in collecting blood from finger prick onto filter paper. The Solomon Island researchers conducted all community consultations, led by HH and JA. The residents of Alasi also agreed to walk for 20-30 minutes from their village on the headland to the closest beach, where they met the team from the Hospital who arrived by motorised canoe. Participants were bled on the beach by torchlight. Researchers from Atoifi Hospital and the College of Nursing performed the survey and prepared filter paper specimens and duplicate sets of blood smears: a set for Atoifi Hospital laboratory and a set for transport to the WHO reference laboratory in Townsville. A team from Atoifi Hospital, led by HH and JA, subsequently informed residents of Alasi and Atoifi of the result of the survey.

## Results

A total of 307 samples were tested, 197 from Alasi and 110 from Atoifi. The samples collected at Atoifi were from people resident on campus and (owing to participation by hospital inpatients) from 12 surrounding villages. Samples were from: Atoifi (80), Gethsemane (1), Gwari (2), Honoa (1), Ilanunu (4), Kwai (4), Kwalakwala (6), Loama (1), Manano (2), Mamulele (1), Na’au (2), Namofaewa (2) and Namolaelae (4). Since the total population of Alasi was 297 and of Atoifi campus 214, the number tested represented 66.3% and 37.4% of the Alasi and Atoifi populations respectively. The latter value included only residents of the Atoifi campus as numerator and denominator, not the hospital inpatients from elsewhere. Overall males comprised 40.7% and adults 76.2%. The Alasi sample had 46.7% males, 68.0% adults and an average age of 25.6 years while the other group had had 30% males, 90.9% adults and an average age of 24.5 years.

One male from Alasi had a positive TropBio Og4C3 test (prevalence overall of 0.33% and for Alasi of 0.51%). This subject was an 18 year old who had lived in Alasi for most of his life and had no clinical evidence of LF. The index case and his wife were seronegative. No microfilaria were found in the blood film from the antigen positive case or in films from 50 antigen negative participants.

## Discussion

**Figure 5:**
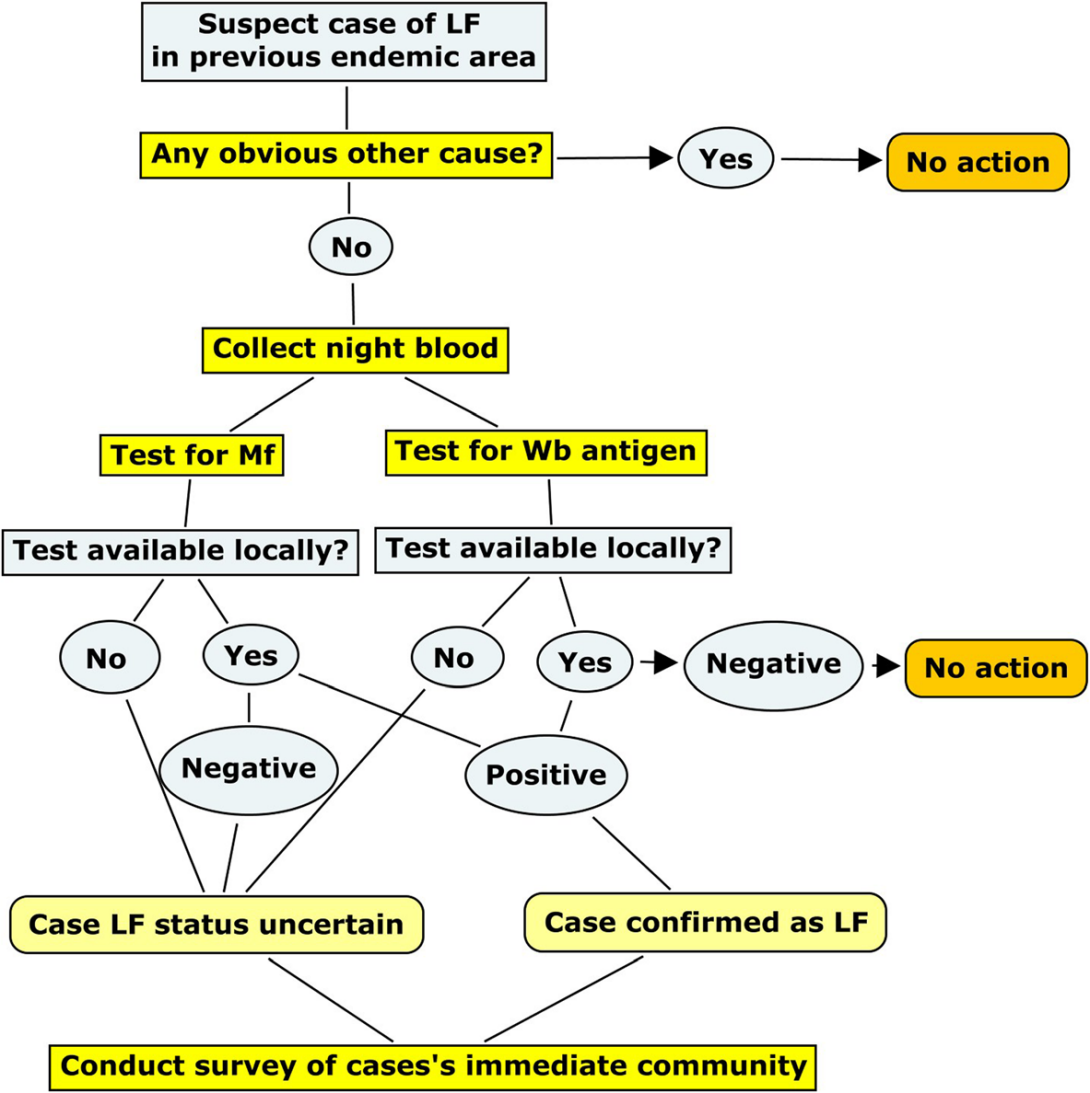
**LF decision tree.** Decision tree for initiation of a survey for LF in a PICT that is in the elimination phase or previously endemic. Wb = *Wuchereria bancrofti.*

The Og4C3 ELISA has a high specificity (95-100%) and positive result indicates a live *W. bancrofti* is present; a false positive is unlikely [20,21]. Although the antigen positive case detected by this survey would have a live worm, since he was microfilaria negative he had probably been infected many years ago. By conducting a comprehensive survey to detect *W. bancrofti* antigen and microfilaria in people living in the local environment of the index case, any possible recent transmission can be detected. In this case there was no evidence of transmission in this area of East Kwaio. This result supports the position that transmission of LF in Solomon Islands has ceased and it is now non-endemic for LF. Such a response to a suspected case of filariasis detected through the primary care system could become a recommended action under the verification process proposed by WHO for countries post-elimination [14]. The decisions that led us to investigate this case have been constructed into a decision tree (Figure 5). This could form the basis of similar investigations of elephantiasis or other manifestations of LF in the PICTs in the elimination phase.

The availability of a group of local health professionals and village leaders trained in research theory and applied research methods allowed the field aspects of the survey to be conducted rapidly (within 1 week of presentation of the index case) and at minimal cost. Specimens were correctly processed and were sent immediately to the international WHO LF reference laboratory in Australia for more tests not available in Solomon Islands. The field survey was funded from the Atoifi Hospital budget and the laboratory costs in Australia from the WHO Collaborating Centre budget. This highlights the importance of sufficient ongoing funding to maintain such a reference centre. Transport of specimens from Atoifi to Townsville was at no cost by an Australian member (DM) of the research workshop returning to Australia. This rapid and very cost effective response highlights the value of having remote health professionals trained in applied research [17]. In addition the local research team felt immense satisfaction in being able to answer local questions of potential national significance [17].

## Conclusions

This model for responding to detection of a new case of lymphoedema is simple and feasible. Field work can be performed by local researchers with minimal resources and laboratory work by a laboratory specialized in LF tests. In countries and regions where LF is eliminated, detecting new cases of elephantiasis or acute lymphoedema of appendages with no obvious alternative cause should be the trigger for a local survey. To be able to do this PICTs eliminating LF should maintain appropriate expertise within their ministries of health / Neglected Tropical Diseases Programme to keep up surveillance, follow up suspected cases and carry out specific specialised tests as required, including maintaining links with the JCU WHO Collaborating Centre for the Control of LF and STH for expert support. Funding to maintain such reference centres for LF control is essential. Finally, we recommend that such events should be recorded in a national database which is then communicated to the global LF programme.

## Competing interests

The authors declare that they have no competing interests.

## Authors’ contributions

HH participated in study design, organizing and conducting the study, analysis, writing and editing; JA participated in study design, organizing and conducting the study, and editing; JG participated in study design and conducting the study; ER participated in study design and conducting the study; RB participated in study design, conducting the study, writing and editing; HJ conducted laboratory tests , interpreted laboratory results, participated in writing and editing; WM participated in study design, interpreted laboratory results, participated in writing and editing; DM participated in study design, organizing and conducting the study, writing and editing; RS participated in study design, conducting the study, performing microscopy, analysis, writing, editing and submitting manuscript. All authors read and approved the final manuscript.
